# Is the content of guidelines/pathways a barrier for the integration of palliative Care in Chronic Heart Failure (CHF) and chronic pulmonary obstructive disease (COPD)? A comparison with the case of cancer in Europe

**DOI:** 10.1186/s12904-017-0243-7

**Published:** 2017-11-28

**Authors:** Naouma Siouta, Karen Van Beek, Sheila Payne, Lukas Radbruch, Nancy Preston, Jeroen Hasselaar, Carlos Centeno, Johan Menten

**Affiliations:** 1 Dept. of Radiation-Oncology and Palliative Medicine, KU Leuven, Leuven, Belgium; 2 Dept. of Radiation-Oncology and Palliative Medicine, University Hospital Gasthuisberg, Leuven, Belgium; 3 International Observatory on End of Life Care Division of Health Research, Faculty of Health and Medicine, Lancaster University, Lancaster, UK; 4 Department of Palliative Medicine, University Hospital of Bonn, Bonn, Germany; 5 Anesthesiology, Pain and Palliative Care, UMC St Radboud, Nijmegen, The Netherlands; 6 Institute for Culture and Society, University of Navarra, Pamplona, Spain

**Keywords:** Care, Palliative, Delivery of health care, Integrated, Medical oncology, Heart failure, Chronic obstructive pulmonary disease

## Abstract

**Background:**

There is a notable inequity in access to palliative care (PC) services between cancer and Chronic Heart Failure (CHF)/Chronic Obstructive Pulmonary Disease (COPD) patients which also translates into discrepancies in the level of integration of PC. By cross-examining the levels of PC integration in published guidelines/pathways for CHF/COPD and cancer in Europe, this study examines whether these discrepancies may be attributed to the content of the guidelines.

**Design:**

A quantitative evaluation was made between integrated PC in published guidelines for cancer and CHF/COPD in Europe. The content of integrated PC in guidelines/pathways was measured using an 11 point integrated PC criteria tool (IPC criteria). A statistical analysis was carried out to detect similarities and differences in the level of integrated PC between the two groups.

**Results:**

The levels of integration between CHF/COPD and cancer guidelines/pathways have been shown to be statistically similar. Moreover, the quality of evidence utilized and the date of development of the guidelines/pathways appear not to impact upon the PC integration in the guidelines.

**Conclusion:**

In Europe, the empirically observed imbalance in integration of PC for patients with cancer and CHF/COPD may only partially be attributed to the content of the guidelines/pathways that are utilized for the PC implementation. Given the similarities detected between cancer and CHF/COPD, other barriers appear to play a more prominent role.

## Background

In Europe, there is an aging population with an increased survival of patients with both malignant and non-malignant diseases and the number of patients in need for palliative care (PC) projected to significantly increase [1, 2]. The substantial number of eligible patients and the complexity of their needs require a more integrated, systematic and sustained approach to the provision of high-quality care.

Integrated PC constitutes a potential unifying framework that enhances PC by integrating it alongside standard treatment that aims to prolong life. More specifically, integrated PC involves bringing together administrative, organisational, clinical and service aspects in order to achieve continuity of care between all those involved in the patient’s care network. It aims to achieve quality of life and a well-supported dying process for the patient and the family in collaboration with all the care givers (paid and unpaid) [3–6]. Importantly, there is evidence-based consensus that integrated PC results in the improvement of the quality of life of patients with both malignant and non-malignant diseases [7–21].

Nonetheless, empirical studies, carried out in a variety of countries, conclusively assert that there is no equity in the access and provision of PC services, [22, 23]. In fact, when compared to patients with cancer, patients with Chronic Heart Failure (CHF) or Chronic Obstructive Pulmonary Disease (COPD) are much less likely to receive PC. Although the exact percentages vary between studies, typically cancer patients have access to PC services at a percentage close to or above 50% whereas the corresponding ones for CHF and COPD are usually much below 25%, sometimes even below 10%, [24–26]. Given that patients with CHF and COPD share the same prevalence of PC-related problems with cancer patients [26–29], this profound imbalance constitutes a critical point of concern. Furthermore, this inequity of access implies that the levels of PC integration for patients with cancer and CHF/COPD are also disparate; this is true even in countries that have been designated as having reached an advanced stage of integration of PC [1, 30, 31].

In practice, the implementation of PC is often based on guidelines or pathways [32]. Guidelines are systematically developed statements to assist practitioners and patient decisions about appropriate health care for specific clinical circumstances. They can be national, international or local. As such, they are often used as a means to reduce variations in treatments within health-care systems, to develop hospital-tailored protocols, to educate students and to assist insurers [32–34]. On the other hand, a care (or clinical) pathway is defined as a complex intervention for mutual decision making and organization of care processes for a well-defined group of patients during a well-defined period [35]*.*

The frequent utilization of guidelines and pathways, combined with the foregoing discussion, suggest that the empirically observed discrepancies with respect to the levels of PC integration might be traced, among others, in the content of the corresponding guidelines/pathways. In this respect, the following hypothesis may be formulated:

*The content of the available guidelines/pathways concerning integration of PC practices is different in cancer and in CHF/COPD and constitutes a barrier for the improvement of the level of PC integration.*

Interestingly, even though the identification of barriers for the improvement of PC integration has attracted considerable attention, [36–38],the validity of the above hypothesis remains, to the best of our knowledge, an open question. A recent systematic review published by the authors, [39], identified existing integrated PC guidelines/pathways for patients with CHF and COPD in Europe. The study revealed, among others, that the level of integration of PC accommodated in existing guidelines/pathways is moderate. Although this finding does suggest that there is still room for improvement, it does not allow for the acceptance or rejection of the aforementioned hypothesis. For a conclusive answer, one needs to juxtapose and analyse existing evidence for CHF and COPD with the corresponding ones for cancer.

The objective of this paper is to examine the validity of the abovementioned hypothesis in Europe. In order to do so, we carry out a comparison and quantitative analysis on the levels of integration of PC between the guidelines/pathways for cancer and CHF/COPD in Europe included in [39, 40].

Before proceeding to the main body of this study, two comments are in order. First. as the notable case of the Liverpool Care Pathway [41] asserts, the completeness of the content of a guideline/pathway does not suffice for its successful implementation. In other words, even if a guideline/pathway has an excellent content on PC integration, its implementation might lead to totally undesirable outcomes. Nevertheless, the completeness of the content does constitute a necessary condition for a successful implementation which gives merit to our hypothesis. Second, in principle, one could directly compare the results from [39, 40] to conclude in favour or against our hypothesis. However, this approach is not well-founded due to the inequality in the number of guidelines/pathways, the differences in the quality of evidence that the guidelines/pathways have been built upon, the differences in the year of publication etc. In other words, a robust assessment of our hypothesis requires the performance of a suitable statistical analysis, like the one employed in the present study.

## Methods

### Comparison and quantitative analysis

In the two systematic reviews [39, 40], the measurement of the level of integration of the PC content of the guidelines was performed via an 11-criteria tool based on the study by [42]. This is a template designed by the American Hospice Foundation Guidelines Committee to provide a practical approach for guideline writers and others to integrate PC into disease management and care services whenever it is relevant. These criteria are described as follows and hereafter shall be referred to as “IPC criteria”.

Integrated Palliative Care (IPC) Criteria.

1. Discussion of illness limitations and prognosis.
2. Recommendations for conducting a whole patient assessment including the patient’s physical, social, psychological, and spiritual issues, their family and community setting.
3. Recommendations for when to make these assessments (referral criteria).
4. Recommendations on when palliative care should be integrated.
5. Assessment of the patient’s goals for care.
6. Continuous goal adjustment as the illness and the person’s disease progresses.
7. Palliative care interventions to reduce suffering as needed.
8. Advance care planning.
9. Recommendation of involving a palliative care team.
10. Recommendations on care during the last hours of living.
11. Recommendations on grief and bereavement care.

Each guideline has been assessed via these IPC criteria and has been assigned the value ‘1’ for each criterion that it fulfils and the value “0”otherwise. By summing up the assigned values we can associate each guideline with a sum score that ranges from “0” to “11”. The range of scores is interpreted on a 12-level Likert scale with zero (0) standing for no integration and eleven [11] standing for utmost integration. It is important to note that these IPC criteria constitute quality indicators for the content of integrated PC in the guidelines and do not provide any information on the efficacy of the implementation into clinical practice of the guidelines which needs to be measured via additional, clinical studies. Moreover, we underline that this assessment tool is yet to be validated. However, and to the best of our knowledge no such validated tool currently exists, which in turn supports our choice.

### Evidence quality assessment

In order to assess the quality of the evidence of the guidelines/pathways in both systematic reviews, the authors employed a 4-level Likert scale presented as follows: a) High Quality Evidence: guidelines/pathways based on both systematic reviews and consensus methods or those developed following the NICE protocol [29], b) Medium Quality Evidence: guidelines/pathways based on systematic review only or based on other types of well referenced evidence, c) Low Quality Evidence: guidelines/pathways based on consensus methods only, d) Very Low Quality Evidence: guidelines/pathways that are unclear (e.g. apparently evidence based but failing to clarify how this was obtained). This quality assessment guide was agreed upon by consensus between the authors and the PC experts of the InSup-C project in the framework of which this study has been performed (http://www.insup-c.eu/).

### Statistical analysis

In order to compare the levels of integration of PC and the levels of the quality of evidence between the guidelines/pathways for cancer and for CHF/COPD statistical significance tests were carried out. Given the ordinal character of the variables, the Mann-Whitney test was utilized at a 95% level of significance (a = 0.05). Correlation analyses, based on the calculation of the Spearman coefficient, were also conducted in order to detect possible correlations between the variables. Additionally, standard descriptive statistics were employed to provide an insight in the general properties of the two groups of guidelines/pathways.

## Results

**Fig. 1:**
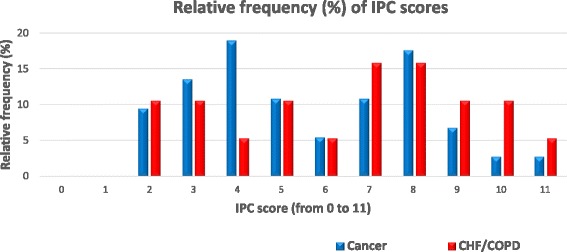
Distribution of Integrated Palliative Care (IPC) scores for cancer and Chronic heart failure/Chronic obstructive pulmonary disease guidelines/pathways. *IPC* Integrated palliative care; *CHF* Chronic heart failure; *COPD* Chronic obstructive pulmonary disease

The number of cancer guidelines was over thrice as high as the one for CHF/COPD (74 vs 19). Table 1 portrays the publication dates of the number of guidelines. The relative frequencies (%) of the IPC scores of the two groups (cancer and CHF/COPD) are depicted in Fig. 1. The medians are M = 5 and M = 7 for cancer and CHF/COPD, respectively.

**Table 1:** Publication dates of the guidelines/pathways

Guidelines/ pathways	Publication Dates
Cancer (*n* = 74)	Date: number of guidelines
	1999: 1
	2002: 1
	2004: 5
	2005: 2
	2006: 2
	2007: 4
	2008: 7
	2009: 4
	2010: 11
	2011: 11
	2012: 12
	2013: 12
	No date available: 2
CHF/COPD (*n* = 19)	2007: 1
	2008: 2
	2010: 8
	2011: 3
	2012: 4
	2013: 1

*CHF* Chronic Heart Failure, *COPD* Chronic Obstructive Pulmonary Disease

The Mann-Whitney test revealed no statistically significant differences between the median IPC scores of the guidelines for cancer and CHF/COPD (U = 573, *p* = 0.19). In other words, the average level of integration of PC in the guidelines for cancer is statistically the same as that for CHF/COPD. The statistical power of the test, corresponding to a large Common Language Effect Size (CLES) = 0.7, was calculated to be approximately equal to 0.8 which is sufficiently large.

**Fig. 2:**
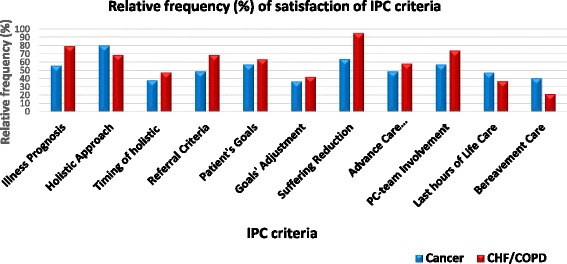
Percentages of cancer and Chronic heart failure/Chronic obstructive pulmonary disease guidelines/pathways satisfying each Integrated Palliative Care (IPC) criterion. *IPC* Integrated palliative care; *CHF* Chronic heart failure; *COPD* Chronic obstructive pulmonary disease

In Fig. 2 the relative frequencies of guidelines scoring on the respective IPC item is reported. In general, the levels of satisfaction are moderate, usually below 60% for both cancer and CHF/COPD. A remarkably low percentage is associated with the 11th criterion that concerns bereavement care.

**Fig. 3:**
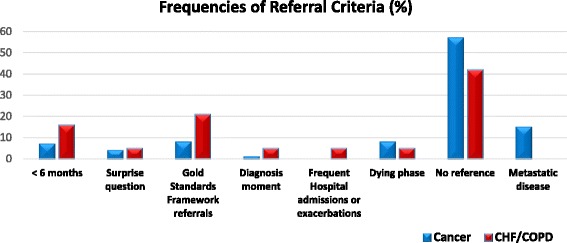
Frequencies of palliative care referral criteria recommended in cancer and Chronic heart failure/Chronic obstructive pulmonary disease guidelines/pathways. *PC* Palliative care; *CHF* Chronic heart failure; *COPD* Chronic obstructive pulmonary disease; Surprise Question = a screening question for physicians “‘Would you be surprised if this patient died in the next year?” that aims to identify end-of-life patients, Gold Standards Framework referrals = see http://www.goldstandardsframework.org.uk/

The different referral criteria recommended by the guidelines/pathways are summarized in Fig. 3. More specifically, Fig. 3 displays the frequencies (%) of the various referral criteria that the guidelines proposed. From this figure, we can infer that only a small number of guidelines/pathways recommended early initiation of PC for either cancer or CHF/COPD.

**Fig. 4:**
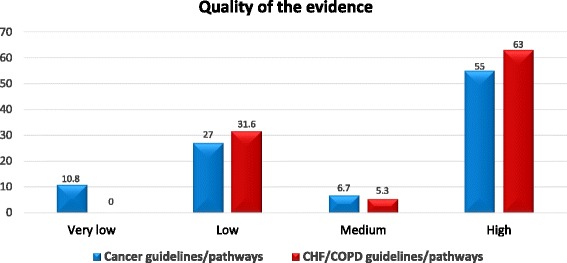
Percentages of cancer and Chronic heart failure/Chronic obstructive pulmonary disease guidelines/pathways meeting each level of quality of evidence. *CHF* Chronic heart failure; *COPD* Chronic obstructive pulmonary disease

The relative frequencies of levels of quality of evidence are portrayed in Fig. 4. The majority of the guidelines/pathways for both cancer and CHF/COPD were of high quality evidence. However, nearly a third of both were of low quality. The Mann-Whitney test showed no statistically significant differences between the medians of quality of evidence level categories between the guidelines/pathways for cancer and CHF/COPD (U = 557.5, *p* = 0.13).

In order to examine whether the statistically observed similarity concerning the levels of integration is biased from the varying levels of quality of evidence, we have performed an additional Mann-Whitney test restricting to guidelines/pathways of high quality. Again, no statistical difference was observed between medians of the samples (U = 328, *p* = 0.08).

We also calculated correlations between the IPC score, the quality of evidence level and the year of publication. The results, based on the calculation of the Spearman coefficient, showed that no statistically significant correlation exists among these various pairs for either cancer or CHF/COPD; see Table 2.

**Table 2:** Correlation analysis between Integrated Palliative Care (IPC) score, quality of evidence and year of publication for cancer and Chronic Heart Failure/Chronic Obstructive Pulmonary Disease guidelines/pathways

		Spearman Co-efficiency	*p*-value
IPC score vs level of quality of evidence	CHF/COPD	0.272	0.259
	Cancer	−0.164	0.162
IPC score vs year of publication	CHF/COPD	0.258	0.206
	Cancer	0.136	0.341
Level of quality of evidence vs year of publication	CHF/COPD	−0.055	0.898
	Cancer	0.005	0.961

*IPC* Integrated Palliative Care, *CHF* Chronic Heart Failure, *COPD* Chronic Obstructive Pulmonary Disease

## Discussion

Our analysis reveals that, at least statistically, there is no difference between the levels of integration of PC in the content of the guidelines/pathways for cancer and CHF/COPD. Moreover, the results remain unaltered even if we confine ourselves to guidelines/pathways that have been built on high quality evidence. This outcome suggests that the content of the guidelines/pathways is inasmuch a barrier for the integration of PC in CHF/COPD as it is for cancer.

Given this statistical equality and the overall moderate level of integration that is observed, it is interesting to examine the satisfaction of the IPC criteria separately. As evidenced in Fig. 2, the trends of the satisfaction of the IPC criteria are also similar. For instance, both groups pay insufficient attention to bereavement care notwithstanding that its importance is well documented [15, 43–45]. Further, one may observe that although the holistic approach is heavily advocated the timing of the holistic assessments is usually not clarified. Finally, it is striking that nearly half of the guidelines/pathways for both groups do not propose specific referral criteria whilst no appreciable consensus was detected among those that did made a recommendation.

The absence of referral criteria from such a high percentage of guidelines in both cancer and CHF/COPD is a perplexing result because it inhibits their efficient implementation by enforcing local services to take initiatives at will. As regards CHF/COPD, it is known that these disease trajectories are quite complex and are typified by interchanging sequences of worsening and partial recovery, with sudden death being a frequent phenomenon [46]. As a consequence, referral criteria for patients with CHF/COPD based on prognostication are quite problematic. On the other hand, the typical trajectory of cancer comprises a gradual decline followed by a short dying phase [47]. Despite the fact that it has been empirically asserted that physicians tend to overestimate the life-expectancy of patients [48–50], referral criteria for cancer based on prognosis can be (and have been) developed based even on international consensus; see, for instance the very recent study of [51]. This, however, is not reflected in our results.

A more striking result concerns the absence of an appreciable correlation between the level of PC integration, the quality of evidence that the guidelines/pathways have been built upon and the year of publication (Table 2). Overall, it suggests that the fact that guidelines/pathways were developed at different times and in different ways (e.g. consensus approaches, systematic review, or expert opinion) is of low importance concerning the content level of PC integration. This is counter-intuitive as one would expect that more recent guidelines/pathways, based on high quality evidence, would perform better in terms of the content of PC integration. A possible explanation for this result is the presence of on-going barriers such as, for example, the determination of referral criteria mentioned above, that inhibit progress in this direction. Moreover, the lack of international consensus on even what is understood by the terminology integrated PC might adversely affect further improvements, as detailed in [12, 18].

Our analysis thus far suggests that the content of PC integration of guidelines/pathways is statistically the same between cancer and CHF/COPD. Furthermore, it does appear to constitute a barrier that is actually comparable in both cases, as evidenced by the individual examination of the criteria. In turn, these point towards the rejection of our research hypothesis. However, one needs to be careful when interpreting the quality of evidence that the guidelines/pathways have been built upon. Indeed, the number of empirical studies (e.g. RCTs) that can provide the basis for guidelines/pathways for cancer are much more than those for CHF/COPD. Consequently, even though two guidelines/pathways may be assessed to be of high quality, in principle, a cancer guideline/pathway may utilize empirical evidence from a larger and more mature basin of studies. Another interesting point concerns the difference in scores above which two guidelines/pathways may be considered to describe appreciable differences in the level of PC integration; this is actually a kind of effect size. In our case setting such a lower limit is actually an ad-hoc procedure. A reasonable option is to partition the 11 IPC criteria as follows: 1,2,3 = low integration, 4,5,6 = medium integration, 7,8,9 = high integration, 10,11 = very high integration. Then a difference of three [3] always moves a guideline/pathway to the next or previous category and thus 3 constitutes a safe choice. Although such have not been observed in the medians of our population one might not exclude their appearance in future relevant studies that will include additional guidelines/pathways e.g. newer ones or outside Europe.

### Limitations

The present study is subject to several limitations. First, the results of this study are limited to Europe and, as such, are not a priori extendable or generalizable to other geographical regions.

Second, a major limitation concerns the tool employed for the measurement of the content of integrated PC. Indeed, the 11-criteria tool employed herein has not been validated in the past. Moreover, some of the criteria are not completely independent; as, for example, reduction of suffering constitutes a necessary, albeit not sufficient, condition for holistic approach that needs to be accounted for separately. However, even though previous studies have documented indicators for the integration of PC [18], to the best of knowledge, no standardized tool for the assessment of PC integration exists in the literature. Consequently, despite the shortcomings of the employed tool, one can still exercise to get, at least, an insight in the problem of interest.

Another limitation could stem from the relatively low number of CHF/COPD guidelines. Even though our sample corresponds to the actual number of the published guidelines/pathways, one might argue that the size of the sample is small. Nevertheless, the statistical power of our study is high enough to capture large effect sizes (CLES = 0.7).

Finally, as expected, the present study inherits all the limitations of the systematic reviews [39, 40] from which it has mined the data.

## Conclusions

The present study has examined whether the content of the guidelines/pathways concerning integration of PC in CHF/COPD constitutes a barrier for the further advancement of PC integration in practice. In order to do so, a comparison and a quantitative evaluation between the corresponding contents in published guidelines/pathways for cancer and CHF/COPD in Europe has been performed. The analysis reveals that content of the levels of integration of PC in patients with cancer and CHF/COPD is statistically the same and it constitutes a barrier that is comparable in both cases.

Despite the limitations of our study, our results have interesting implications. They suggest that, although the content of published guidelines/pathways is a factor that impedes the further integration of PC, it cannot solely justify the remarkable inequity in access of PC between patients with cancer and CHF/COPD. Such imbalances require barriers of higher gravity; such as perceptions of the role of PC for patients with chronic disease or gaps in relevant PC education.

## Abbreviations

CHF: Chronic Heart Failure
COPD: Chronic Obstructive Pulmonary Disease
IPC: Integrated Palliative Care
PC: Palliative care
